# Supplementary material for: A data-driven approach to violin making
Source: Sci Rep. 2021 May 4;11:9455. doi: 10.1038/s41598-021-88931-z (PMC8096844; doi:10.1038/s41598-021-88931-z)
Supplement: Supplementary file 1 — Supplementary Information. [file 41598_2021_88931_MOESM1_ESM.pdf]

Supplementary Material for ‘A Data-Driven Approach to Violin Making’

Sebastian Gonzalez[1,*], Davide Salvi[1], Daniel Baeza[2], Fabio Antonacci[1], and Augusto Sarti[1]

[1]Musical Acoustics Lab at the Violin Museum of Cremona, DEIB - Politecnico di Milano, Cremona Campus, Italy
[2]Department of Electrical Engineering, Faculty of Physical and Mathematical Sciences, University of Chile, Chile
[*]tsuresuregusa@gmail.com

ABSTRACT

This document contains equations referenced in the main text and examples of the top plates created in the dataset.

Parametric creation of a violin outline

Implementation of Stradivari’s parametric outline. Equations defining the 17 points and 20 parameters used. p_i stands for x, y points whereas alphabetic letters are one dimensional parameters corresponding to radii and apperture angles. We start from the point p_0 and define iteratively points either on the circular arc (e.g. p_1 or p_2) or on the radii of an existing circunference (e.g. p_3 or p_4). Points $p_1$5 and $p_1$6 are found by imposing that they lay in the symmetry axis hency their formula is slightly different. To understand each parameter graphically please refer to Fig. 1.

```
p0  = {-x0, 0};
p1  = p0 + aa {Cos[a], Sin[a]};
p2  = p0 + aa {Cos[b], Sin[b]};
p3  = p2 - c {Cos[b], Sin[b]};
p4  = p1 - cc {Cos[a], Sin[a]};
p5  = p4 + cc {Cos[d], Sin[d]};
p6  = p3 + c {Cos[e], Sin[e]};
p7  = p6 + f {Cos[g], Sin[g]};
p8  = p5 + h {Cos[d2], Sin[d2]};
p9  = p8 + h {Cos[l], Sin[l]};
p10 = p9 + k {Cos[l], Sin[l]};
p11 = p10 +  k {Cos[\[Pi]/2 + hh], Sin[\[Pi]/2 + hh]};
p12 = p11 +  k {Cos[hh], Sin[hh]};
p13 = p7 + f {Cos[gg], Sin[gg]};
p14 = p13 + ff {Cos[gg], Sin[gg]};
p15 = p14 + ff {Cos[-\[Pi]/2 - kk], Sin[-\[Pi]/2 - kk]};
p16 = {0, p15[[2]] + rr2 Cos[ArcSin[Sin[kk] ff/rr2 - p14[[1]]/rr2]]};
p17 = {0, p11[[2]] - rr Cos[ArcSin[Sin[hh] k/rr - p10[[1]]/rr]]};
```

Interpolation of the thickness profile

The algorithm to interpolate between different thickness regions has been described in detail in[1], however we explain here the idea behind the interpolation method. First, the individual regions are assigned different thicknesses. Then, in between the regions a linear interpolation is used to connect the different regions. Finally, a smoothing algorithm in 2D is used to eliminate any sharp thickness variation, in accordance with actual practice of violin making. See Fig. 2 for a 1D diagram of the process. It must be noted that the edge is a constant thickness of 2.7mm

Examples of top plates

Figure 3 shows three examples of the violin top plates contained in the dataset. Only the middle example can be considered a "typical" violin shape, the other other two probably do not correspond to actual historical examples of violins.

References

1. Gonzalez, S., Salvi, D., Antonacci, F. & Sarti, A. Eigenfrequency optimisation of free violin plates. *The J. Acoust. Soc. Am.* **149**, 1400–1410 (2021).

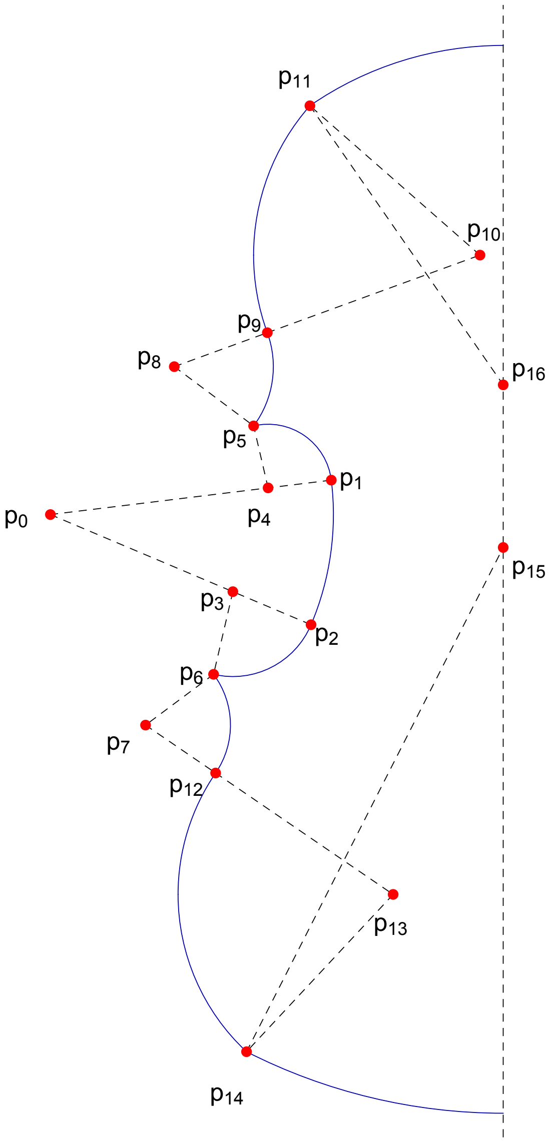

Figure 1. Diagram showing the construction method for the violin outline. We start by defining the p_0, then p_1 and p_2 are defined as two points on the circle centred in p_0 of radius `aa` and angles `a` and `b` respectively. Only half of the outline is shown since our model is symmetric

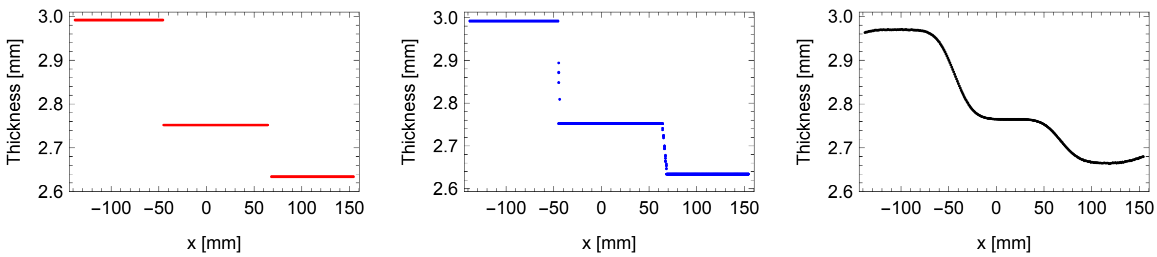

Figure 2. Schematic of the three steps of the interpolation and homogenisation process between thickness areas of the violin top plate: first, each elliptical region has a discrete height, left. Then, a linear interpolation is used between the regions, centre. Finally, to smooth the transitions in a way analogous to actual violin making practice, the hard steps are smoothed with smoothing function as in[1]. Note that this is a process in 2D in the actual plates, this is just a 1D visualisation.

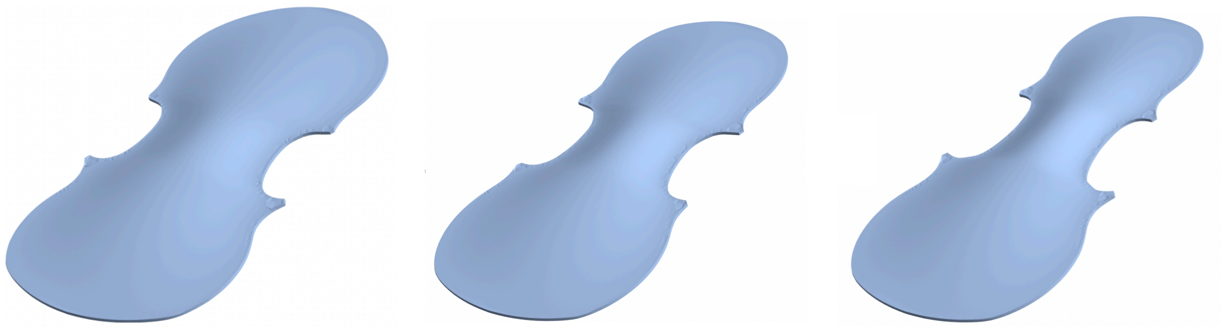

Figure 3. Three examples of the violin top plates created with our algorithm, from left to right decreacing the width of the violin.
